# GOLPH3 predicts survival of colorectal cancer patients treated with 5-fluorouracil-based adjuvant chemotherapy

**DOI:** 10.1186/1479-5876-12-15

**Published:** 2014-01-21

**Authors:** Zaozao Wang, Beihai Jiang, Lei Chen, Jiabo Di, Ming Cui, Maoxing Liu, Yiyuan Ma, Hong Yang, Jiadi Xing, Chenghai Zhang, Zhendan Yao, Nan Zhang, Bin Dong, Jiafu Ji, Xiangqian Su

**Affiliations:** 1 Key laboratory of Carcinogenesis and Translational Research (Ministry of Education), Department of Minimally Invasive Gastrointestinal Surgery, Peking University Cancer Hospital & Institute, 52 Fucheng Road, Haidian District, 100142 Beijing, China; 2 Key laboratory of Carcinogenesis and Translational Research (Ministry of Education), Department of Pathology, Peking University Cancer Hospital & Institute, 100142 Beijing, China; 3 Key laboratory of Carcinogenesis and Translational Research (Ministry of Education), Department of Surgery, Peking University Cancer Hospital & Institute, 100142 Beijing, China

**Keywords:** GOLPH3, 5-fluorouracil (5-FU), Colorectal cancer (CRC), Chemotherapy, Prognosis

## Abstract

**Background:**

Golgi phosphoprotein 3 (GOLPH3) has been validated as a potent oncogene involved in the progression of many types of solid tumors, and its overexpression is associated with poor clinical outcome in many cancers. However, it is still unknown the association of GOLPH3 expression with the prognosis of colorectal cancer (CRC) patients who received 5-fluorouracil (5-FU)-based adjuvant chemotherapy.

**Methods:**

The expression of GOLPH3 was determined by qRT-PCR and immunohistochemistry in colorectal tissues from CRC patients treated with 5-FU based adjuvant chemotherapy after surgery. The association of GOLPH3 with clinicopathologic features and prognosis was analysed. The effects of GOLPH3 on 5-FU sensitivity were examined in CRC cell lines.

**Results:**

GOLPH3 expression was elevated in CRC tissues compared with matched adjacent noncancerous tissues. Kaplan-Meier survival curves indicated that high GOLPH3 expression was significantly associated with prolonged disease-free survival (DFS, *P* = 0.002) and overall survival (OS, *P* = 0.011) in patients who received 5-FU-based adjuvant chemotherapy. Moreover, multivariate analysis showed that GOLPH3 expression was an independent prognostic factor for DFS in CRC patients treated with 5-FU-based chemotherapy (HR, 0.468; 95%CI, 0.222-0.987; *P* = 0.046). *In vitro*, overexpression of GOLPH3 facilitated the 5-FU chemosensitivity in CRC cells; while siRNA-mediated knockdown of GOLPH3 reduced the sensitivity of CRC cells to 5-FU-induced apoptosis.

**Conclusions:**

Our results suggest that GOLPH3 is associated with prognosis in CRC patients treated with postoperative 5-FU-based adjuvant chemotherapy, and may serve as a potential indicator to predict 5-FU chemosensitivity.

## Background

Colorectal cancer (CRC) is the third most common cancer worldwide and the second leading cause of cancer deaths in Europe and North America [1,2]. The 5-year survival rate of CRC has been improved over the past two decades because of progress in early diagnosis and treatment modalities [1-3]. Currently, the usage of fluorouracil-based adjuvant chemotherapy after resection of CRC is considered as a major therapeutic approach in prolonging patient survival; and the standard first-line treatment for CRC is the combination therapy of 5-fluorouracil (5-FU) with DNA-damaging agent oxaliplatin [2-4]. However, a proportion of patients fail to benefit from adjuvant chemotherapy, either as a result of drug resistance or due to chemotherapy-induced toxicity [4-6]. Consequently, there is an urgent need to identify prognostic factors and markers that can predict chemotherapy sensitivity or resistance in order to select patients that most likely to be benefit from the adjuvant chemotherapy before treatment.

Golgi phosphoprotein 3 (GOLPH3) was initially identified as a Golgi membrane protein. It is highly conserved in a range of organisms from yeast to humans and plays a crucial role in Golgi trafficking and morphology [7-9]. Recently it has been validated as a potent oncogene frequently targeted for copy number gain at chromosome 5p13 in several human cancers, including colorectal cancer [10]. Also, it has been reported that GOLPH3 overexpression promotes cell proliferation and tumorigenesis by activating mTOR signaling, enhancing AKT activity, as well as decreasing FOXO1 transcriptional activity [10,11]. Importantly, the ability of GOLPH3 to modulate rapamycin-induced tumor cell death identifies it as a potential positive predictor of rapamycin sensitivity in tumor therapy [10]. Moreover, high expression of GOLPH3 has been shown to be associated with poor prognosis in breast cancer, esophageal squamous cell carcinoma (ESCC), oral tongue cancer, gastric cancer, prostate cancer, glioblastoma multiforme, gliomas, and rhabdomyosarcoma[11-18] . However, the clinical relevance of GOLPH3 in patients with CRC remains largely unknown.

In the current study, we examined the expression of GOLPH3 in CRC in order to determine its correlation with clinical characteristics and prognosis. Moreover, we investigate the potential association between 5-FU chemosensitivity of CRC cells and GOLPH3 level. Our results suggest that increased expression of GOLPH3 correlates with favourable prognosis in patients treated with 5-FU-based adjuvant chemotherapy and predicts higher 5-FU sensitivity in CRC cells. Taken together, our results suggest that GOLPH3 may serve as a potential indicator to predict 5-FU chemosensitivity.

## Methods

### Patients and tissue specimens

This retrospective study included 130 CRC patients with available tumour specimens who received surgical resection followed by 5-FU-based post-operative adjuvant chemotherapy from January 2004 to December 2006, in Peking University Cancer Hospital & Institute. Patients treated with pre-operative radiotherapy or adjuvant chemotherapy were excluded from this study. A total of 130 CRC tissues and 75 matched non-cancerous tissues were obtained from resected tumors and adjacent tissues, respectively. Then, all the tissues were fixed in buffered formalin and embedded in paraffin. The CRC and normal tissues were all pathologically confirmed. TNM stages of patients were determined according to the American Joint Committee on Cancer (AJCC) classification guidelines. The clinicopathological characteristics of the patient cohort are summarized in Table 1. For all patients, combination chemotherapy of oxaliplatin, 5-FU, with leucovorin was started within 4 to 8 weeks after surgery, according to their recovery. The treatment cycle was repeated every 2 weeks for 12 cycles. All patients were followed until August 2012. The median follow-up period was 62 months. Another 30 CRC tissues, as well as the matched adjacent noncancerous tissues were fresh-frozen tissues stored at −80°C for quantitative reverse transcription-PCR (qRT-PCR) analysis. The collection of tissue samples was approved and supervised by the Research Ethics Committee of Peking University Cancer Hospital & Institute. Written informed consents were obtained from all patients prior to surgery.

**Table 1:** Association between GOLPH3 expression and clinicopathological variables of CRC patients treated with 5-FU-based adjuvant chemotherapy

**Variables**	**Cases**	**GOLPH3 expression**		***P***
		**Low**	**High**	
Age				
<60 yr	58	32	26	0.718
≥60 yr	72	42	30	
Gender				
Female	50	21	29	**0.007**
Male	80	53	27	
Tumor location				
Colon	104	62	42	0.215
Rectum	26	12	14	
Tumor size				
≤4 cm	43	24	19	0.858
>4 cm	87	50	37	
Depth of Invasion				
T1/T2	13	7	6	0.813
T3/T4	117	67	50	
Lymph node metastasis				
Negative	50	24	26	0.104
Positive	80	50	30	
Distant metastasis				
Negative	105	56	49	0.090
Positive	25	18	7	
TNM stage				
II	45	21	24	0.086
III/IV	85	53	32	
Histological type				
Adenocarcinoma	121	70	51	0.498
Mucinous	9	4	5	
Tumor differentiation				
Well	17	10	7	0.812
Moderate	87	49	38	
Poor	17	11	6	
Unknown	9			
Recurrence				
Negative	83	42	41	**0.006**
Positive	42	32	10	
Unknown	5			
Survival				
Alive	82	40	42	**0.014**
Dead	48	34	14	

*P* values in bold were statistically significant.

### Quantitative reverse transcription-PCR (qRT-PCR)

Total RNA from colorectal tissues or CRC cells were extracted using Trizol (Invitrogen, Carlsbad, CA, USA). The isolated total RNA was transcribed into cDNA using a reverse transcription kit (Promega, Madison, WI, USA) according to the manufacturer’s instructions. The synthesized cDNA was used as templates in qRT-PCR to evaluate the relative mRNA levels of GOLPH3 and GAPDH (as internal control) using primers summarized in Additional file 1: Table S1. The primers were conjungated with SYBR Green PCR Master Mix (Toyobo Co. Ltd., Osaka, Japan) and the PCR was performed with the ABI 7500 real-time PCR system (Life Technologies, Carlsbad, California, USA). Data were analysed using ABI 7500 V 2.0.6 software and presented in terms of relative quantification (RQ) to GAPDH, based on calculations of 2^-∆Ct^ where ∆Ct = Ct (Target) -Ct (Reference). Fold change was calculated by the 2 ^-∆∆Ct^ method [19]. Each sample was examined in triplicate.

### Immunohistochemistry

Paraffin-embedded CRC specimens were sliced into 4 μm sections. Immunohistochemical staining was performed as described [20] with the primary rabbit polyclonal antibody against GOLPH3 (Cat#: 19112-1-AP; 1:400, Proteintech). The visualization signal was developed with diaminobenzidine (Sigma). Sections were counterstained with hematoxylin. Tissue staining with purified IgG from normal rabbit serum was used as a negative control. GOLPH3 staining was evaluated by two experienced pathologists, BD and ZL, independently without any knowledge of the clinical data. The total GOLPH3 immunostaining score was calculated both as the percentage of positive cells, and the intensity of cytoplasmic staining: The proportion of positive tumor cell was scored as: 0, no positive tumor cells; 1, 1%–10% positive tumor cells; 2, 11%–35% positive tumor cells; 3, 36%–70% positive tumor cells; and 4, >70% positive tumor cells. Staining intensity was scored as: 0, no staining; 1, weak staining (light yellow); 2, moderate staining (yellow brown); and 3, strong staining (brown). The staining index for GOLPH3 expression in colorectal cancer lesions was calculated by multiplying the two scores obtained for each sample and obtained values of 0, 1, 2, 3, 4, 6, 9, or 12 [13]. A minimal measurement of score six was predetermined as high GOLPH3 expression.

### Cell lines and cell culture

Human CRC cell lines SW480, RKO, LoVo, HT29, and HCT116 were purchased from American Type Culture Collection (ATCC, Manassas, VA). Human CRC cell lines SW480, RKO and HT29 were grown in RPMI-1640 (Gibco), while LoVo and HCT116 were cultured in DMEM medium (Gibco). All the media were supplemented with 10% fetal bovine serum (FBS, PAA) and antibiotics (100 units/mL penicillin and 100 μg/mL streptomycin) at 37°C in a humidified incubator with 5% CO_2_.

### Plasmid and siRNA transfections

The GOLPH3 expressing plasmid (pCMV-Myc-GOLPH3) was constructed by subcloning a PCR product encoding full-length human GOLPH3 cDNA into the pCMV-Myc plasmid. The GOLPH3-targeting siRNA and the negative control siRNA were designed and purchased from Shanghai Gene Pharma (Shanghai, China). The primer sequences used for subcloning, sequences of GOLPH3 siRNA and the control were summarized in Additional file 1: Table S1. RKO and LoVo cells were transiently transfected with the pCMV-Myc-GOLPH3 plasmid or siRNAs using Lipofectamine™ 2000 (Invitrogen) according to the manufacturer’s protocol. Transfected cells were incubated for 24 h before further treatment.

### Methylthiazolyldiphenyl-tetrazolium bromide (MTT) assay

MTT assay was used to determine cell sensitivity to 5-FU. RKO and LoVo cells transfected with GOLPH3 expressing plasmid or GOLPH3 siRNA were seeded 4000 cells/well in 96-well plates and incubated at 37°C for 24 h. Then the medium was replaced with 150 μL of fresh medium containing 5-FU of 0, 12.5, 25, 50, 100 and 200 μM. After 48 h exposure, MTT dye was added to each well with the final concentration of 5 mg/mL, and then the cells were incubated for another 4 h at 37°C. Before measurement, the resultant formazan crystals were dissolved by replacing the culture medium with equal volume of dimethylsulfoxide (DMSO). The spectrometric absorbance at 570 nm was measured with a microplate reader (Bio-Rad, USA). The inhibition ratio was calculated as [(OD value of control - OD value of the sample)/OD value of control] × 100%. The experiment was repeated three times.

### Apoptosis analysis

Apoptosis was determined using the PI/Annexin V dual staining kit (Biosea Biotechnology, Beijing, China) according to the manufacturer’s instructions. Briefly, 5 × 10^5^ cells were plated in each well of six-well plates and incubated at 37°C for 24 h. After being transfected with GOLPH3 expressing plasmid or GOLPH3 siRNA, the CRC cells were then exposed to 0 or 200 μM of 5-FU, and incubated for 48 h. The cells were then harvested and stained with AnnexinV/fluorescein isothiocyanate (FITC) and propidium iodide (PI) and subjected to flow cytometry analysis using FACSAria (BD, Franklin Lakes, NJ).

### Western blot

After transfection, RKO and LoVo cells were exposed to 0, 100 and 200 μM of 5-FU or 0, 2 and 20 μM of paclitaxel. After 48 h incubation, cells were harvest and total protein (100 μg) isolated from cells were electrophoresed followed by electrotransferring onto a nitrocellulose membrane. The expression of proteins was detected using primary antibody against GOLPH3 (Cat#: 19112-1-AP; 1:500; Proteintech), PARP (Cat#: 9542; 1:1000; Cell Signaling Technology), and β-actin (Cat#: TA-09; 1:1000; ZSGB-BIO). The signal was detected by the ECL Western blot detection kit (Amersham, Little Chalfont, UK). Band intensity was quantified using NIH Scion Image software and normalized to β-actin.

### Statistical analysis

Statistical analysis was performed using SPSS software (version 13.0, SPSS, Inc., Chicago, IL). Differences in mRNA expression between tumor samples and the matched adjacent noncancerous tissue samples were evaluated using paired-sample t-tests. Pearson’s chi square and Fisher’s exact tests were used to analyse the correlation between GOLPH3 expression and clinicopathological characteristics. Survival was determined using the Kaplan-Meier methods and compared with the log-rank test. Univariate and multivariate survival analyses were performed using the Cox proportional hazard regression model. Two-sided values of *P* < 0.05 were considered statistically significant.

## Results

### GOLPH3 expression and association with clinicopathological characteristics in CRC

**Figure 1:**
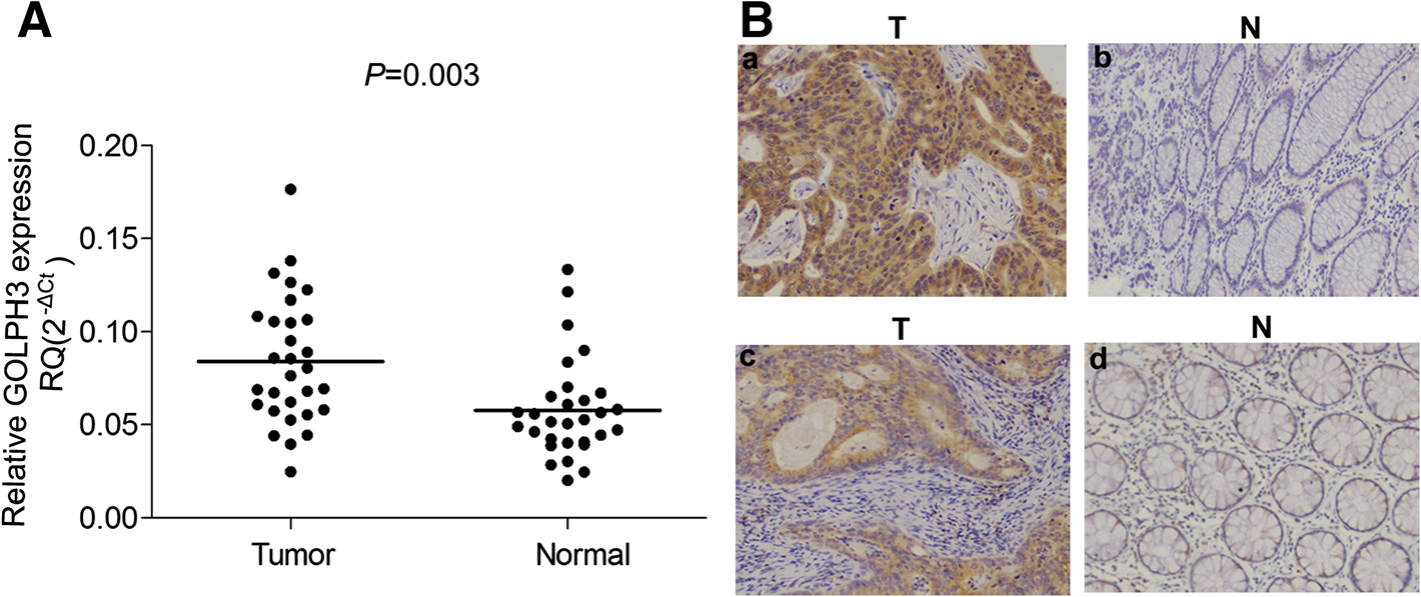
**Expression of GOLPH3 in colorectal tissues. (A)** GOLPH3 mRNA level in CRC tissues (Tumor) and matched adjacent noncancerous tissues (Normal) was detected by qRT-PCR. The relative expression of GOLPH3 was normalized with GAPDH mRNA level. **(B)** Representative immunohistochemical images indicate strong (a) or moderate (c) GOLPH3 staining in CRC tissues (T), and (b and d) staining of GOLPH3 in matched noncancerous tissues adjacent to tumors (N). Magnification is 200 × .

In this study, we examined GOLPH3 mRNA levels in matched cancerous and adjacent noncancerous colorectal tissues from 30 CRC patients. qRT-PCR results showed that GOLPH3 transcripts were significantly elevated in cancerous tissues than in matched adjacent noncancerous tissues (*P* = 0.003, Figure 1A). Moreover, we measured GOLPH3 protein expression by immunohistochemistry in 130 cases. Consistent with mRNA expression, high GOLPH3 expression was detected in 56 out of 130 (43.1%) CRC tissues, compared with 10 out of 75 (13.3%) in matched adjacent noncancerous tissues (*P* < 0.0001) (Figure 1B). Then we investigated possible correlations between GOLPH3 expression and CRC clinicalpathological characteristics. Our data showed that GOLPH3 expression was correlated with gender (*P* = 0.007), recurrence (*P* = 0.006) and survival (*P* = 0.014). However, no significant associations were found between GOLPH3 expression and other clinicopathological features (Table 1).

### Association between GOLPH3 protein expression and survival of patients treated with 5-FU-based adjuvant chemotherapy

**Figure 2:**
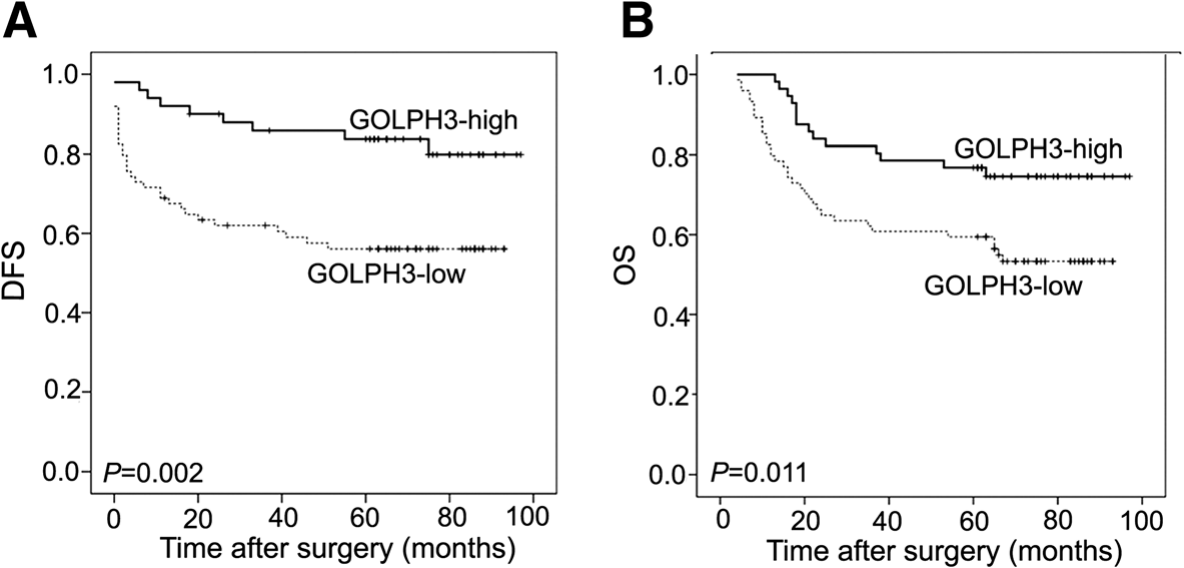
**Kaplan-Meier curves for DFS and OS.** High levels of GOLPH3 expression was significantly associated with favourable DFS **(A)** and OS **(B)** in patients treated with 5-FU-based adjuvant chemotherapy.

Kaplan-Meier survival curves and log-rank test survival analysis were used to determine the prognostic value of GOLPH3 on survival of patients who received 5-FU-based adjuvant chemotherapy. The results showed that patients with tumors exhibiting high GOLPH3 expression had substantially longer disease-free survival (DFS; *P* = 0.002, Figure 2A) and overall survival (OS; *P* = 0.011, Figure 2B) than those with tumors exhibiting low GOLPH3 expression. These data suggest that high GOLPH3 expression is associated with favourable prognosis in patients treated with 5-FU adjuvant chemotherapy.

Furthermore, Cox proportional hazard regression analysis was performed to evaluate the prognostic significance of clinicopathological parameters. Univariate analysis showed that high GOLPH3 expression was significantly associated with longer DFS (HR, 0.334; 95%CI, 0.159-0.702; *P* = 0.004, Table 2) and OS (HR, 0.458; 95%CI, 0.245-0.853; *P* = 0.014, Additional file 2: Table S2) in patients treated with 5-FU-based adjuvant chemotherapy. All factors that showed prognostic significance in the univariate analysis were included in the multivariate analysis. Multivariate analysis showed that GOLPH3 appeared to be an independent prognostic factor for prolonged DFS (HR, 0.468; 95% CI, 0.222-0.987; P = 0.046, Table 2). However, patients with high levels of GOLPH3 expression retained only a borderline significant association with favourable OS compared to those with low levels of GOLPH3 expression (HR, 0.557; 95%CI, 0.292-1.062; P = 0.076, Additional file 2: Table S2).

**Table 2:** Univariate and multivariate analysis of GOLPH3 in patients who received 5-FU-based chemotherapy with respect to DFS

**Variables**	**Univariate**		***P***	**Multivariate**		***P***
	**HR**	**95% CI**		**HR**	**95% CI**	
Age (≥60 yr vs <60 yr)	1.037	0.583−1.844	0.901			
Gender (Male vs Female)	2.318	1.179−4.556	**0.015**	2.011	0.980−4.128	0.057
Tumor location (Rectum vs Colon )	0.958	0.476−1.929	0.904			
Tumor size (>4 cm vs ≤4 cm)	1.424	0.751−2.699	0.279			
TNM stage (III/IV vs II)	11.545	3.578−37.253	**<0.0001**	29.406	4.031−214.527	**<0.0001**
Histological type (Mucinous Vs Adenocarcinoma)	1.086	0.337−3.501	0.89			
Tumor differentiation (Poor/moderate vs Well)	2.794	0.865−9.025	0.086			
GOLPH3 expression (High vs Low)	0.334	0.159−0.702	**0.004**	0.468	0.222−0.987	**0.046**

*HR* hazard ratio, *CI* confidence interval, *P* values in bold were statistically significant.

### GOLPH3 regulates 5-FU-induced cytotoxicity in CRC cells

**Figure 3:**
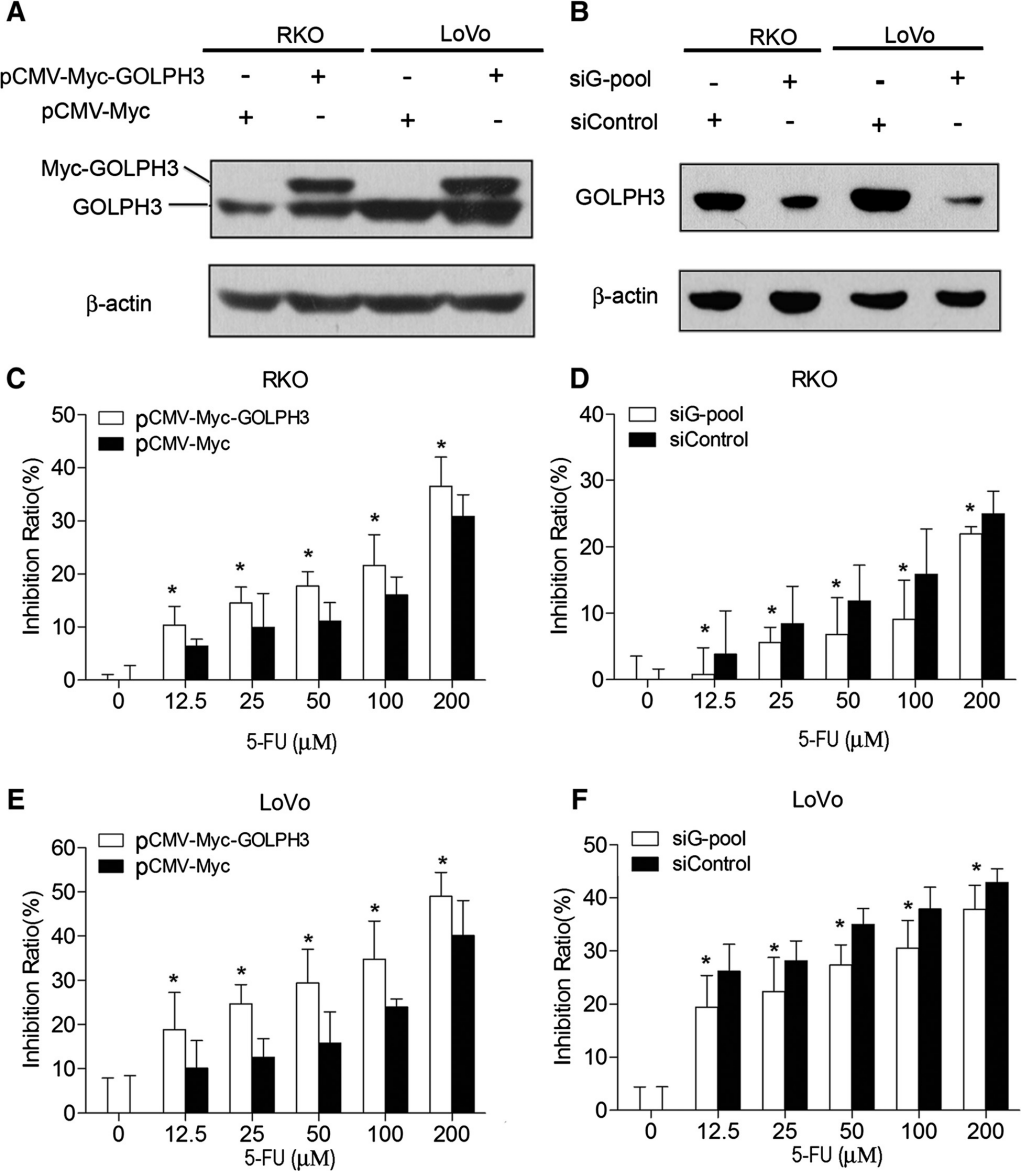
**GOLPH3 regulates 5-FU sensitivity in CRC cells. (A and B)** Western blot analysis of GOLPH3 expression in RKO and LoVo cells transfected with plasmid encoding full length of GOLPH3 or GOLPH3 siRNA pool (siG-pool). β-actin was used as a loading control. Cells were treated with different concentrations of 5-FU (0, 12.5, 25, 50, 100, 200 μM). And cell viability was analyzed by MTT assay. The growth inhibition ratio increased in GOLPH3-overexpressing CRC cells **(C and E)** and decreased in GOLPH3-silencing CRC cells **(D and F)** in a 5-FU dose-dependent manner. The values are mean ± SD of three independent experiments, *P < 0.05 compared with control cells.

Then we examined the contribution of GOLPH3 expression to 5-FU sensitivity *in vitro*. First, we measured the expression of GOLPH3 in CRC cell lines, including SW480, RKO, LoVo, HT29 and HCT116. qRT-PCR and Western blot analysis showed that GOLPH3 was ubiquitously expressed in all of five CRC cell lines at both mRNA and protein levels (Additional file 3: Figure S1). The highest GOLPH3 expression was detected in LoVo cells while RKO cells showed the lowest GOLPH3 expression. Accordingly, RKO and LoVo cells were used to perform the following experiments *in vitro*. Secondly, to facilitate further study, we used either plasmid encoding full length of GOLPH3 or GOLPH3 siRNAs to either overexpress or knockdown GOLPH3 levels in CRC cells, respectively. Western blot analysis confirmed the efficiency of GOLPH3 overexpression and knockdown in CRC cells (Figure 3A and 3B). Since siRNA pool (siG-pool, a combination of three independent siRNAs including siG-1, siG-2 and siG-3) could achieve stronger on-target gene knockdown with minimal off-target effect (Additional file 4: Figure S2) [21], we used siG-pool to silence GOLPH3 expression in CRC cells.

We further evaluated the effects of GOLPH3 expression on the 5-FU cytotoxicity to RKO and LoVo cells using MTT analysis. As shown in Figure 3C and 3E, overexpression of GOLPH3 significantly increased 5-FU-induced cytotoxicity in a dose-dependent manner in both RKO and LoVo cells. Moreover, knockdown of GOLPH3 dramatically decreased 5-FU-induced cell death and increased 5-FU resistance in these cells (*P* < 0.05, Figure 3D and 3F). Our results suggest that GOLPH3 played an important role in CRC cell sensitivity to 5-FU.

### GOLPH3 modulates 5-FU sensitivity through regulation of 5-FU-induced apoptosis

**Figure 4:**
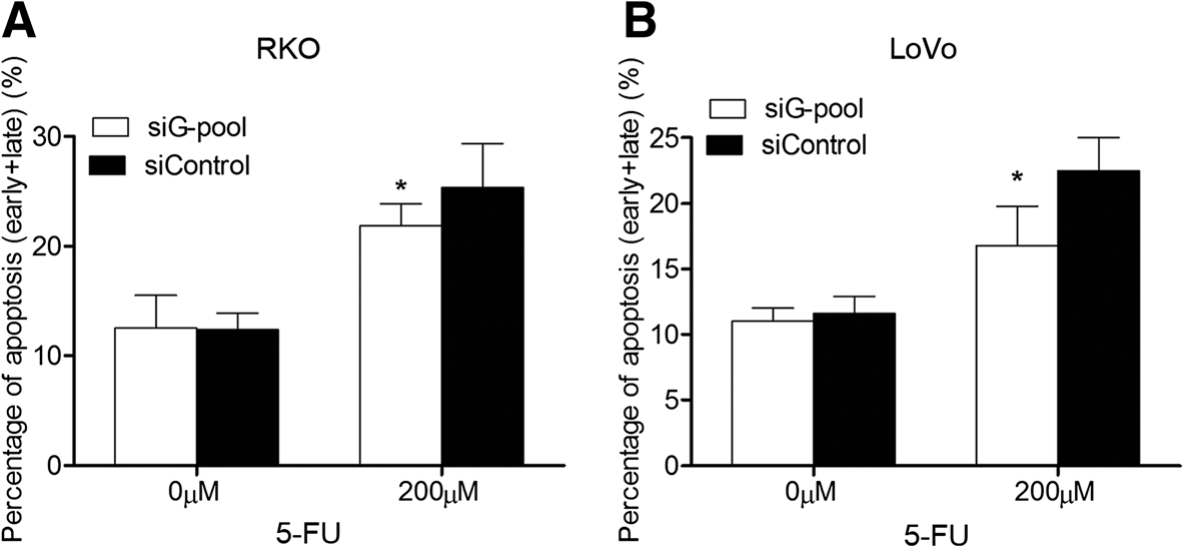
**Knockdown of GOLPH3 inhibited 5-FU-induced apoptosis in CRC cells.** RKO **(A)** and LoVo **(B)** cells were transfected with GOLPH3 siRNA pool (siG-pool) or control siRNA (siControl), and then treated with 5-FU of 0 or 200 μM for 48 h. Apoptosis was detected by flow cytometry using Annexin-V-FITC and propidium iodide (PI) dual labelling. Data are presented as percentage of early and late apoptotic cells of total number of cells examined. All experiments were performed in triplicate. The values are mean ± SD, *P < 0.05.

The effect of GOLPH3 on chemosensitivity was confirmed by analysing 5-FU-induced apoptosis. Flow cytometric analysis showed that 5-FU-induced apoptosis was significantly reduced in GOLPH3-silencing RKO and LoVo cells (Figure 4 and Additional file 5: Figure S3), and increased in GOLPH3-overexpressing cells, compared with control cells (Additional file 6: Figure S4), suggesting that GOLPH3 could sensitize 5-FU-induced apoptosis.

**Figure 5:**
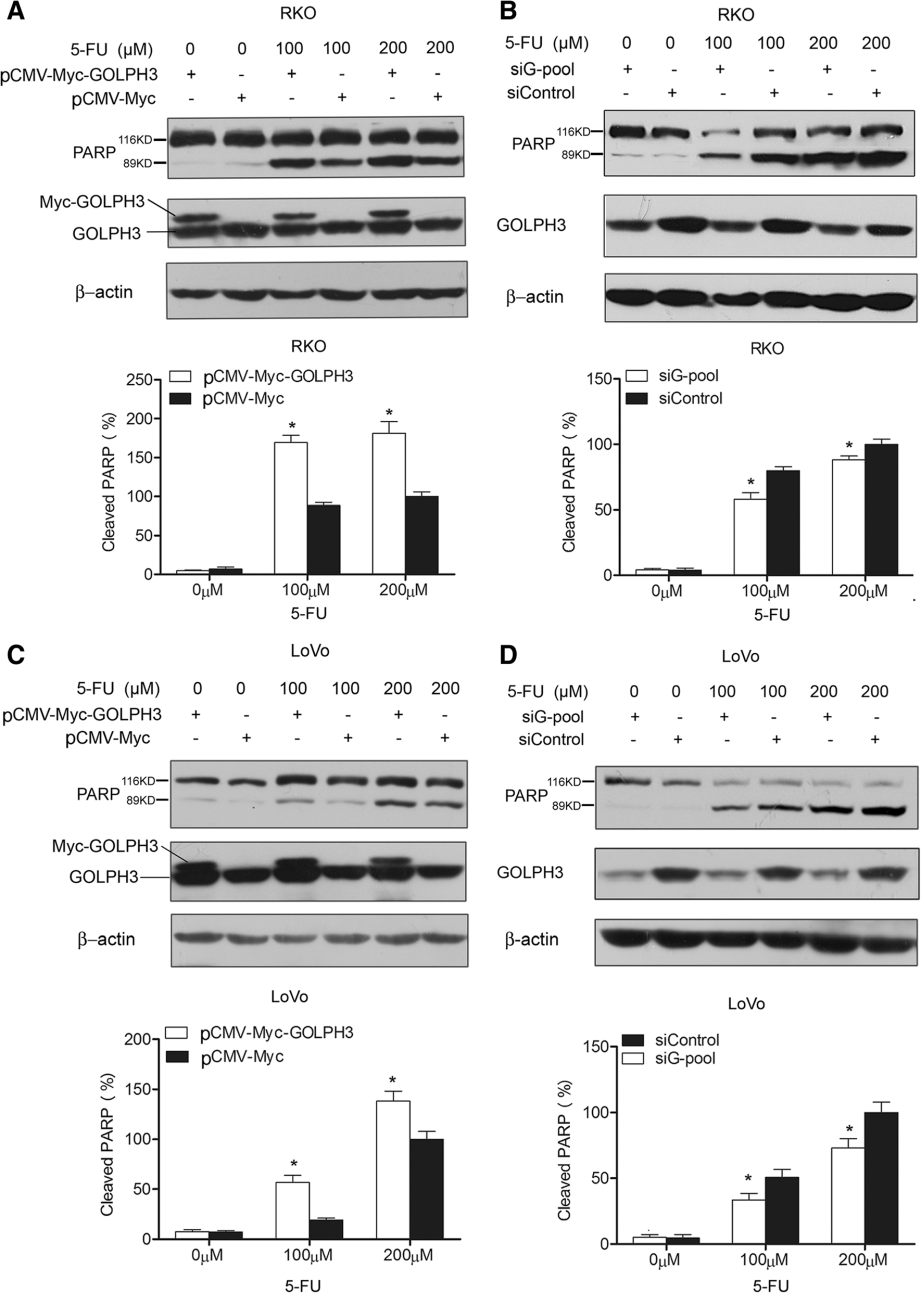
**GOLPH3 regulated 5-FU-induced apoptosis via modulation of PARP cleavage.** RKO and LoVo cells were transfected with pCMV-Myc-GOLPH3/pCMV-Myc (GOLPH3 over-expression, **A and C**) or GOLPH3 siRNA pool (siG-pool)/control siRNA (siControl) (GOLPH3 silencing, **B and D**) and then treated with indicated concentrations of 5-FU (0, 100, 200 μM) for 48 h. Western blot analysis showed the alteration of cleaved PARP and GOLPH3. Upper panels, one representative of three similar Western blot analysis. Lower panels, relative protein ratio were normalized to β-actin. All data are mean ± SD, *P < 0.05.

Moreover, to further confirm the effect of GOLPH3 on 5-FU-induced apoptosis, changes in cleaved PARP were detected by Western blot. PARP is a substrate of caspase-3 and −7 which cleave it into 89 and 24 kDa fragments. Cleavage of PARP is one of the classic markers for activation of downstream signals during apoptosis [22]. Although the expression of GOLPH3 changed minimally in CRC cells treated with increased doses of 5-FU (Additional file 7: Figure S5), overexpression of GOLPH3 increased the cleaved PARP in both RKO and LoVo cells administrated with 5-FU (Figure 5A and Figure 5C). Moreover, knockdown of GOLPH3 by siRNA reduced the cleavage of PARP in both cells under 5-FU treatment (Figure 5B and Figure 5D). These results suggest that overexpression of GOLPH3 sensitizes cells to 5-FU-induced apoptosis, whereas down-regulation of GOLPH3 protects cells against 5-FU-induced apoptosis.

## Discussion

In the present study, we demonstrated that GOLPH3 was highly expressed in CRC tissues compared with matched adjacent noncancerous tissues. High levels of GOLPH3 expression were associated with prolonged survival in CRC patients treated with postoperative 5-FU-based adjuvant chemotherapy. And multivariate analysis identified GOLPH3 as an independent prognostic factor for DFS. Furthermore, we found that overexpression of GOLPH3 could facilitate the cytotoxicity of 5-FU to CRC cells, while knockdown of GOLPH3 hindered the sensitivity of CRC cells to 5-FU-induced apoptosis *in vitro*. These findings suggested that GOLPH3 modulates 5-FU sensitivity and may serve as a potential indicator to predict 5-FU chemosensitivity.

GOLPH3, which is a component of the Golgi matrix, has been identified as a novel protooncogene by Scott et al. since 2009 [10]. Recent clinical studies have indicated that high levels of GOLPH3 expression promote tumorigenesis and progression of several types of malignancies, and correlates with poor survival in various cancers [10-16]. In colon adenocarcinoma, it has been revealed that GOLPH3 is amplified at the 5p13 region [10]. It has been showed that tumors with high levels of GOLPH3 were significantly more sensitive to rapamycin treatment *in vivo*[10]. These findings suggest that GOLPH3 may associate with tumor cell sensitivity to anticancer agents. 5-FU-based adjuvant chemotherapy is a standard treatment for patients with stage III to IV CRC, and stage II CRC with a high risk of recurrence [2-4]. Whether GOLPH3 plays a role in predicting the therapeutic effect of 5-FU for individual patient is our concern. Thus, in this study, we investigated the clinical significance of GOLPH3 in patients treated with 5-FU-based adjuvant chemotherapy, and the potential influence of GOLPH3 on adjuvant chemotherapy. Our study revealed GOLPH3 expression was elevated in CRC tissues compared with matched adjacent noncancerous tissues. However, in contrast with other studies, our results showed that high levels of GOLPH3 expression indicated favourable prognosis in patients who underwent 5-FU-based adjuvant chemotherapy, suggesting that CRC patients with tumors exhibiting high GOLPH3 expression may be more likely to benefit from 5-FU-based adjuvant chemotherapy than those with tumors exhibiting low GOLPH3 expression. Possible explanations for these disparate findings between our study and others may include different genetic features of patients, different types of cancer or variable tumor stages, variable doses and schedules of adjuvant chemotherapy among previous studies, and limited amount of patients in some studies. Moreover, although many oncogenes are correlated with cell resistance to chemotherapy, there are still some oncogenes which could enhance chemotherapeutic sensitivity and are correlated with prolonged survival [23-28]. For example, XB130 has been reported to promote tumor cell proliferation, inhibit apoptosis and increase cell invasion in esophageal squamous cell carcinoma, lung and thyroid cancer [23-25]. However, in gastric cancer, patients with higher XB130 expression were found to have a better prognosis. Meanwhile, patients with upregulated XB130 were more sensitive to 5-FU and have a longer survival time [26]. Likewise, Fra-1 and ID1 have been proved to exert oncogenic function in breast and prostate cancers, whereas overexpression of these genes not only predict better survival in cancer patients, but also sensitized cancer cells to different chemotherapeutics [27,28]. Similarly, although GOLPH3 is a potent oncogene, GOLPH3-expressing tumors were more sensitive to rapamycin [10], as well as 5-FU in our study. Accordingly, patients with high levels of GOLPH3 will be more sensitive to 5-FU and have prolonged survival.

Since GOLPH3 plays critical roles in activating mTOR signalling and conferring increased sensitivity to mTOR inhibitor, rapamycin, whether GOLPH3 expression level predicts sensitivity to chemotherapy agents, such as 5-FU, need to be elucidated. In this study, we found that GOLPH3 overexpression sensitized CRC cells to 5-FU-induced apoptosis *in vitro*. These results raise the possibility that the effects of GOLPH3 on cellular chemosensitivity contributes to the role of GOLPH3 in predicting survival of CRC patients who received 5-FU-based adjuvant chemotherapy. Similarly, the chemosensitivity of CRC cells to paclitaxel, a chemotherapy agent widely used in advanced gastrointestinal carcinoma, was also found to be modulated by GOLPH3 (Additional file 8: Figure S6).

Although more precise biochemical basis and mechanisms for GOLPH3 in modulating the sensitivity to cytotoxic drugs remains to be explored, our results indicated that GOLPH3 may serve as a positive predictor of 5-FU sensitivity. To further validate our findings, prospective randomized clinical studies for patients with or without 5-FU treatment are required. Moreover, for patients with recurrent or metastatic CRC, Response Evaluation Criteria in Solid Tumors (RECIST) guidelines should be applied to evaluate the tumor response and the clinical benefit from 5-FU-based chemotherapy more precisely.

## Conclusions

In conclusion, our study revealed that GOLPH3 expression could predict the clinical outcome in CRC patients treated with postoperative 5-FU-based adjuvant chemotherapy. Patients with tumors exhibiting high GOLPH3 expression were more likely to benefit from 5-FU-based adjuvant chemotherapy than those with tumors exhibiting low GOLPH3 expression. Functionally, we showed that GOLPH3 affected the sensitivity of CRC cells to 5-FU-induced cytotoxity. Taken together, GOLPH3 may serve as a potential indicator to predict 5-FU chemosensitivity.

## Competing interests

The authors declare that they have no competing interests.

## Authors’ contributions

XS conceived and designed the study. ZW, BJ, LC and JD performed the experiments. ZW, BJ and JD analyzed the data. MC, ML, YM, HY, JX, CZ ZY, NZ, BD and JJ contributed reagents, materials, and analysis tools. BJ and ZW wrote the paper. All authors read and approved the final manuscript.

## Supplementary Material

Additional file 1: Table S1 Primers and sequences. Click here for file

Additional file 2: Table S2 Univariate and multivariate analysis of GOLPH3 in patients who underwent 5-FU-based chemotherapy with respect to OS. Click here for file

Additional file 3: Figure S1 Expression of GOLPH3 in CRC cell lines. GOLPH3 mRNA (A) and protein expression (B) in CRC cell lines (SW480, RKO, LoVo, HT29, and HCT116) were determined by qRT-PCR and Western blot, respectively. SW480 was used as a calibrator in ∆∆Ct analysis for qRT-PCR. Click here for file

Additional file 4: Figure S2 Efficiency of siRNAs-mediated knockdown of GOLPH3 in RKO cells. Knockdown of GOLPH3 was evaluated in RKO cells transfected with GOLPH3 siRNAs (siG-1, siG-2, siG-3 or siG-pool) or control siRNA (siControl) using (A) qRT-PCR and (B) Western blot. SiControl was used as a calibrator in ∆∆Ct analysis for qRT-PCR. Click here for file

Additional file 5: Figure S3 Influence of GOLPH3 silencing on 5-FU induced CRC cell apoptosis. Fraction of apoptotic cells were analysed by flow cytometry in siGOLPH3-transfected RKO (A) and LoVo cells (B) cultured with or without 200 μM of 5-FU. The population of apoptotic cell was calculated as the percentages of cells in the upper- and lower-right quadrants. All experiments were performed in triplicate. Click here for file

Additional file 6: Figure S4 Overexpression of GOLPH3 sensitized 5-FU-induced apoptosis in RKO cells in a dose-dependent manner. (A) RKO cells were transfected with pCMV-Myc-GOLPH3 or control (pCMV-Myc), and then treated with 5-FU of 0, 200 or 400 μM for 48 h. Apoptosis was detected by flow cytometry using Annexin-V-FITC and propidium iodide (PI) dual labelling. (B) Data are presented as percentage of early and late apoptotic cells of total number of cells examined. All experiments were performed in triplicate. The values are mean ± SD, *P < 0.05. Click here for file

Additional file 7: Figure S5 5-FU treatment does not affect the protein level of GOLPH3. GOLPH3 expression was determined by Western blot in RKO (A) and LoVo cells (B) treated with different concentrations of 5-FU. Click here for file

Additional file 8: Figure S6 The expression of GOLPH3 and cleaved PARP in RKO cells treated with paclitaxel. (A) Expression of GOLPH3 was determined by Western blot in RKO cells treated with various concentrations of paclitaxel for 48 h. Paclitaxel treatment does not affect GOLPH3 expression. (B) Knockdown of GOLPH3 reduced paclitaxel-induced apoptosis. The cleavage of PARP in paclitaxel treated GOLPH3-silencing and control cells was examined by Western blot. The results are representative of three independent experiments. Click here for file
