# Supplementary material for: GOLPH3 predicts survival of colorectal cancer patients treated with 5-fluorouracil-based adjuvant chemotherapy
Source: J Transl Med. 2014 Jan 21;12:15. doi: 10.1186/1479-5876-12-15 (PMC4029222; doi:10.1186/1479-5876-12-15)
Supplement: Additional file 1: Table S1 — Primers and sequences. [file 1479-5876-12-15-S1.docx]

**Additional file 1: Table S1. Primers and sequences**

|  | **Sequences** |
| --- | --- |
| **Primers for subcloning:** |  |
| GOLPH3 forward primer | 5’-CCGGAATTCGGACCATGACCTCGCTGACCCAGCGC-3’ |
| GOLPH3 reverse primer | 5’-CCGCTCGAGTTACTTGGTGAACGCCGCCAC-3’ |
| **qRT-PCR primers：** |  |
| GOLPH3 forward primer | 5’-GCCTCCAGAAACGGTCCAG-3’ |
| GOLPH3 reverse primer | 5’-GTCAATACACCCTTTTCCACCA-3’ |
| GAPDH forward primer | 5’-TGCACCACCACCTGCTTAGC-3’ |
| GAPDH reverse primer | 5’-GGCATGGACTGTGGTCATGAG-3’ |
| **siRNA:** |  |
| siGOLPH3-1 (siG-1) sense | GCCUCAUCAAGAAAGUACATT |
| siGOLPH3-1 (siG-1) antisense | UGUACUUUCUUGAUGAGGCTT |
| siGOLPH3-2 (siG-2) sense | GUCCAGAACUGGAUUGAAUTT |
| siGOLPH3-2 (siG-2) antisense | AUUCAAUCCAGUUCUGGACTT |
| siGOLPH3-3 (siG-3) sense | GGCUGUAUGUUAAUUGAAUTT |
| siGOLPH3-2 (siG-3) antisense | AUUCAAUUAACAUACAGCCTT |
| control siRNA sense | UUCUCCGAACGUGUCACGUTT |
| control siRNA antisense | ACGUGACACGUUCGGAGAATT |
