# Supplementary material for: GOLPH3 predicts survival of colorectal cancer patients treated with 5-fluorouracil-based adjuvant chemotherapy
Source: J Transl Med. 2014 Jan 21;12:15. doi: 10.1186/1479-5876-12-15 (PMC4029222; doi:10.1186/1479-5876-12-15)
Supplement: Additional file 2: Table S2 — Univariate and multivariate analysis of GOLPH3 in patients who underwent 5-FU-based chemotherapy with respect to OS. [file 1479-5876-12-15-S2.docx]

**Additional File 2: Table S2**. **Univariate and multivariate analysis of GOLPH3 in patients who underwent 5-FU-based chemotherapy with respect to OS**

| Variables | Univariate | |  | Multivariate | |  |
| --- | --- | --- | --- | --- | --- | --- |
|  | HR | 95%CI | *P* | HR | 95%CI | *P* |
| Age  (≥60 yr vs <60 yr) | 1.451 | 0.835-2.520 | 0.187 |  |  |  |
| Gender  (Male vs Female) | 1.617 | 0.901-2.901 | 0.107 |  |  |  |
| Tumor location  (Rectum vs Colon ) | 0.614 | 0.290-1.302 | 0.204 |  |  |  |
| Tumor size  (>4cm vs ≤4cm) | 1.602 | 0.872-2.946 | 0.129 |  |  |  |
| TNM stage  (III/IV vs II) | 8.971 | 3.235-24.880 | **<0.0001** | 12.844 | 3.084-53.490 | **<0.0001** |
| Histological type  (Mucinous vs Adenocarcinoma) | 1.228 | 0.443-3.401 | 0.693 |  |  |  |
| Tumor differentiation (Poor/moderate vs Well) | 3.225 | 1.003-10.365 | **0.049** | 1.871 | 0.576-6.070 | 0.297 |
| GOLPH3 expression  (High vs Low) | 0.458 | 0.245-0.853 | **0.014** | 0.557 | 0.292-1.062 | 0.076 |

*HR* hazard ratio, *CI* confidence interval, *P* values in bold were statistically significant.
