# Supplementary figures and images for: GOLPH3 predicts survival of colorectal cancer patients treated with 5-fluorouracil-based adjuvant chemotherapy
Source: J Transl Med. 2014 Jan 21;12:15. doi: 10.1186/1479-5876-12-15 (PMC4029222; doi:10.1186/1479-5876-12-15)

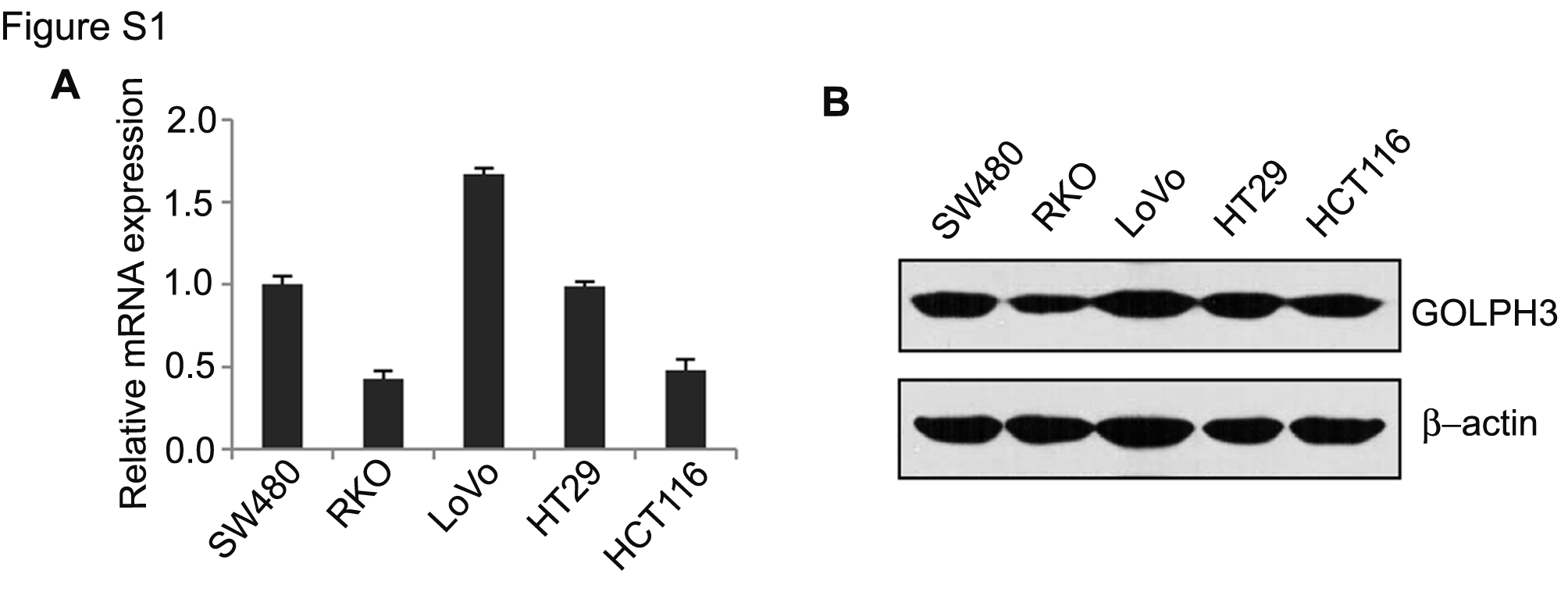

Supplement: Additional file 3: Figure S1 — Expression of GOLPH3 in CRC cell lines. GOLPH3 mRNA (A) and protein expression (B) in CRC cell lines (SW480, RKO, LoVo, HT29, and HCT116) were determined by qRT-PCR and Western blot, respectively. SW480 was used as a calibrator in ∆∆Ct analysis for qRT-PCR. [file 1479-5876-12-15-S3.tiff]

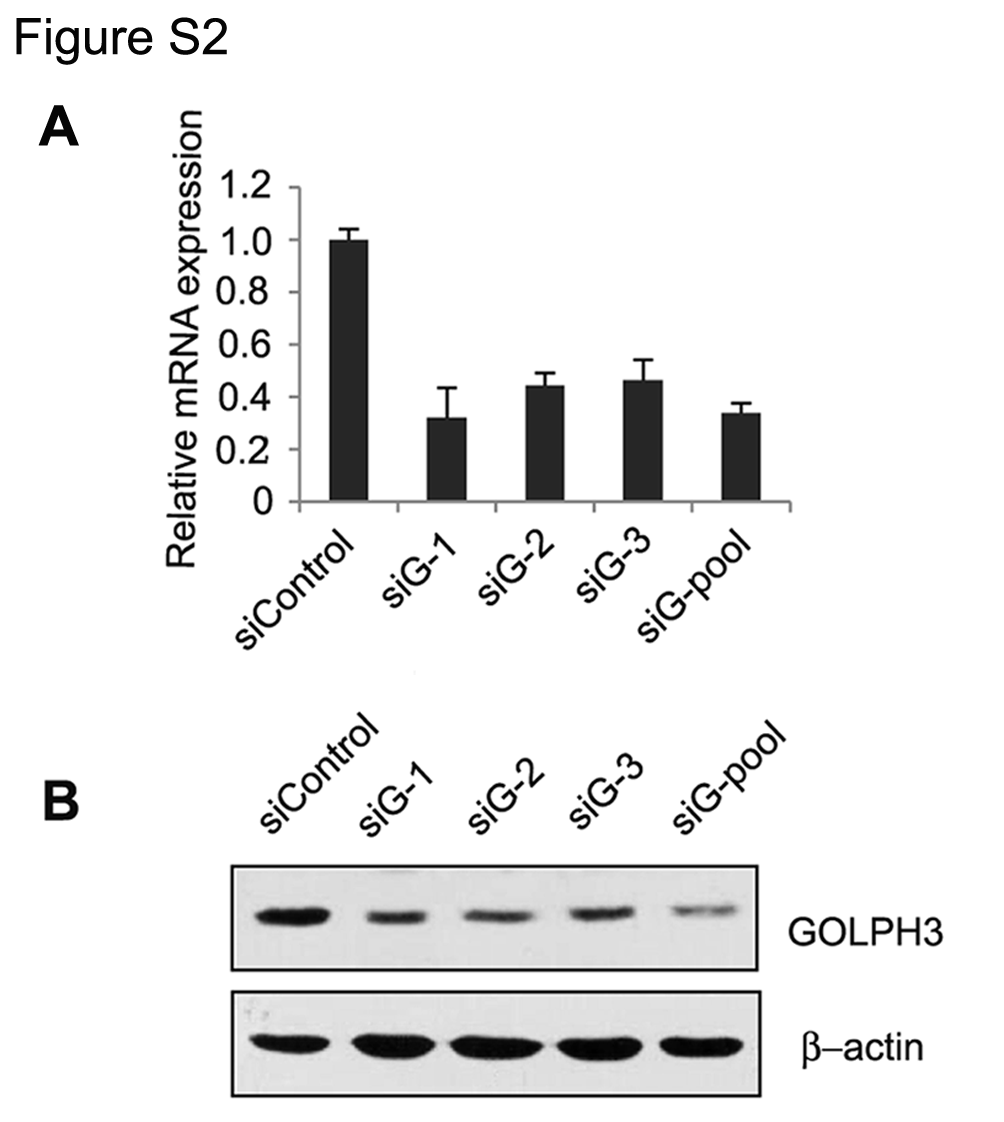

Supplement: Additional file 4: Figure S2 — Efficiency of siRNAs-mediated knockdown of GOLPH3 in RKO cells. Knockdown of GOLPH3 was evaluated in RKO cells transfected with GOLPH3 siRNAs (siG-1, siG-2, siG-3 or siG-pool) or control siRNA (siControl) using (A) qRT-PCR and (B) Western blot. SiControl was used as a calibrator in ∆∆Ct analysis for qRT-PCR. [file 1479-5876-12-15-S4.tiff]

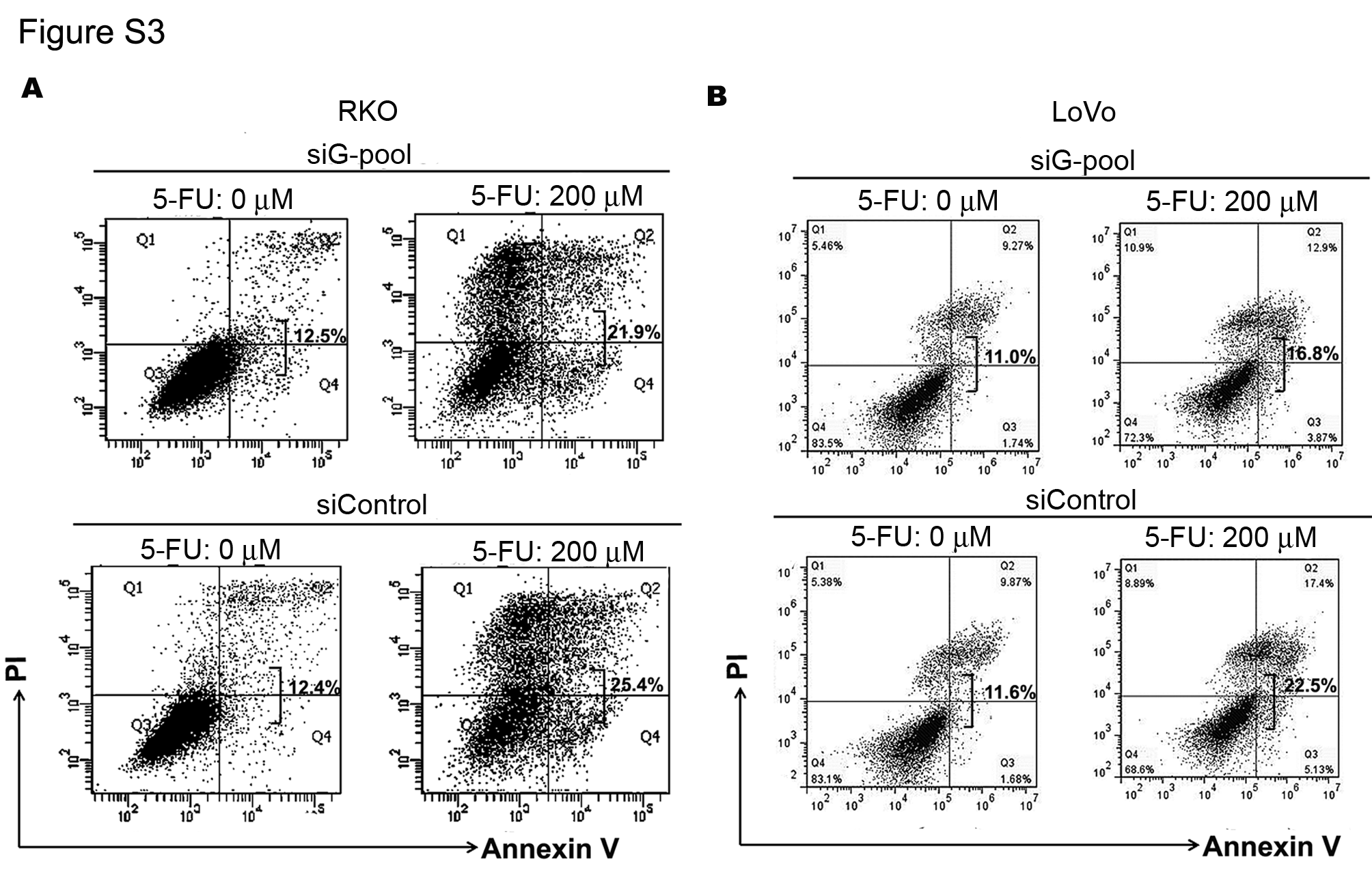

Supplement: Additional file 5: Figure S3 — Influence of GOLPH3 silencing on 5-FU induced CRC cell apoptosis. Fraction of apoptotic cells were analysed by flow cytometry in siGOLPH3-transfected RKO (A) and LoVo cells (B) cultured with or without 200 μM of 5-FU. The population of apoptotic cell was calculated as the percentages of cells in the upper- and lower-right quadrants. All experiments were performed in triplicate. [file 1479-5876-12-15-S5.tiff]

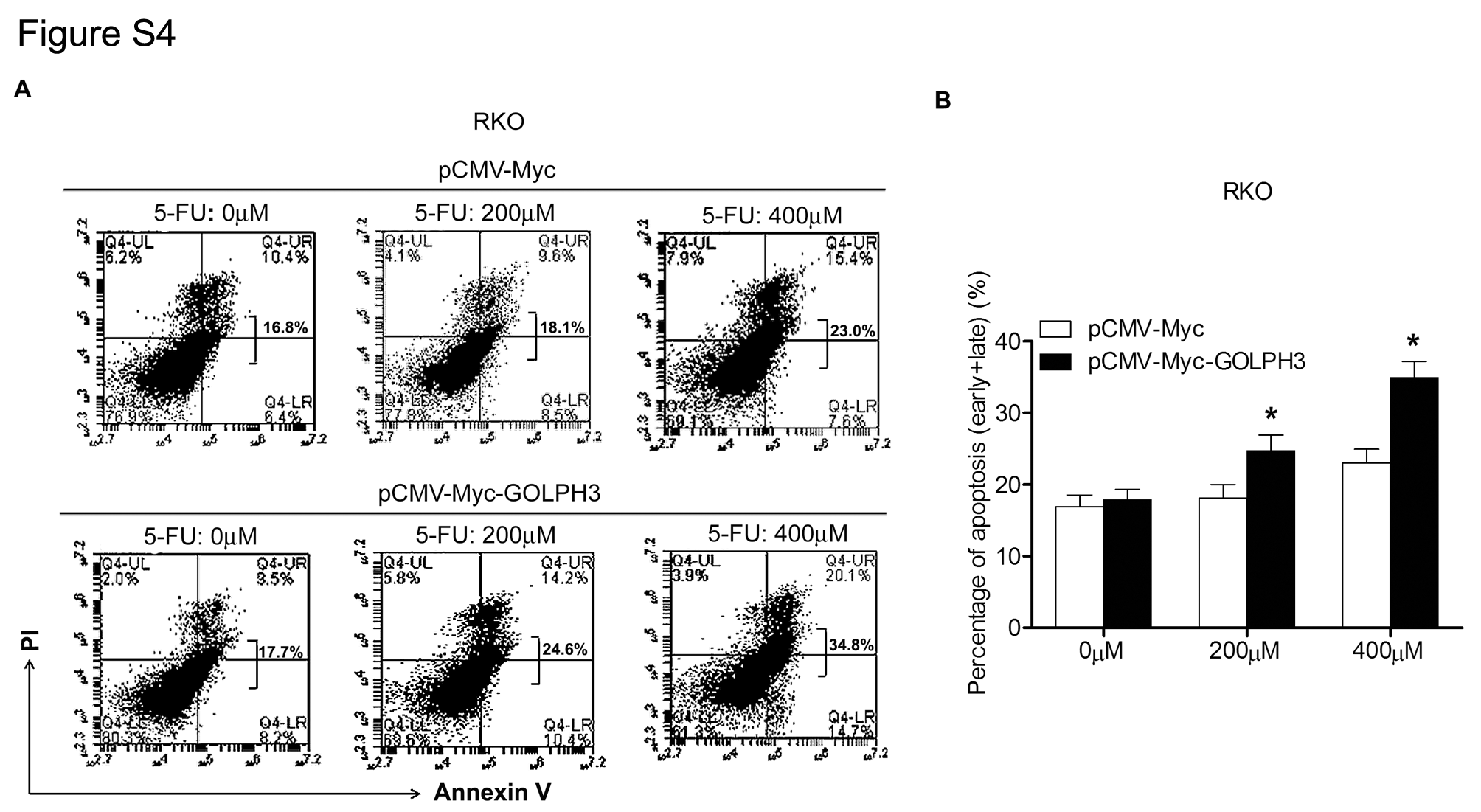

Supplement: Additional file 6: Figure S4 — Overexpression of GOLPH3 sensitized 5-FU-induced apoptosis in RKO cells in a dose-dependent manner. (A) RKO cells were transfected with pCMV-Myc-GOLPH3 or control (pCMV-Myc), and then treated with 5-FU of 0, 200 or 400 μM for 48 h. Apoptosis was detected by flow cytometry using Annexin-V-FITC and propidium iodide (PI) dual labelling. (B) Data are presented as percentage of early and late apoptotic cells of total number of cells examined. All experiments were performed in triplicate. The values are mean ± SD, *P < 0.05. [file 1479-5876-12-15-S6.tiff]

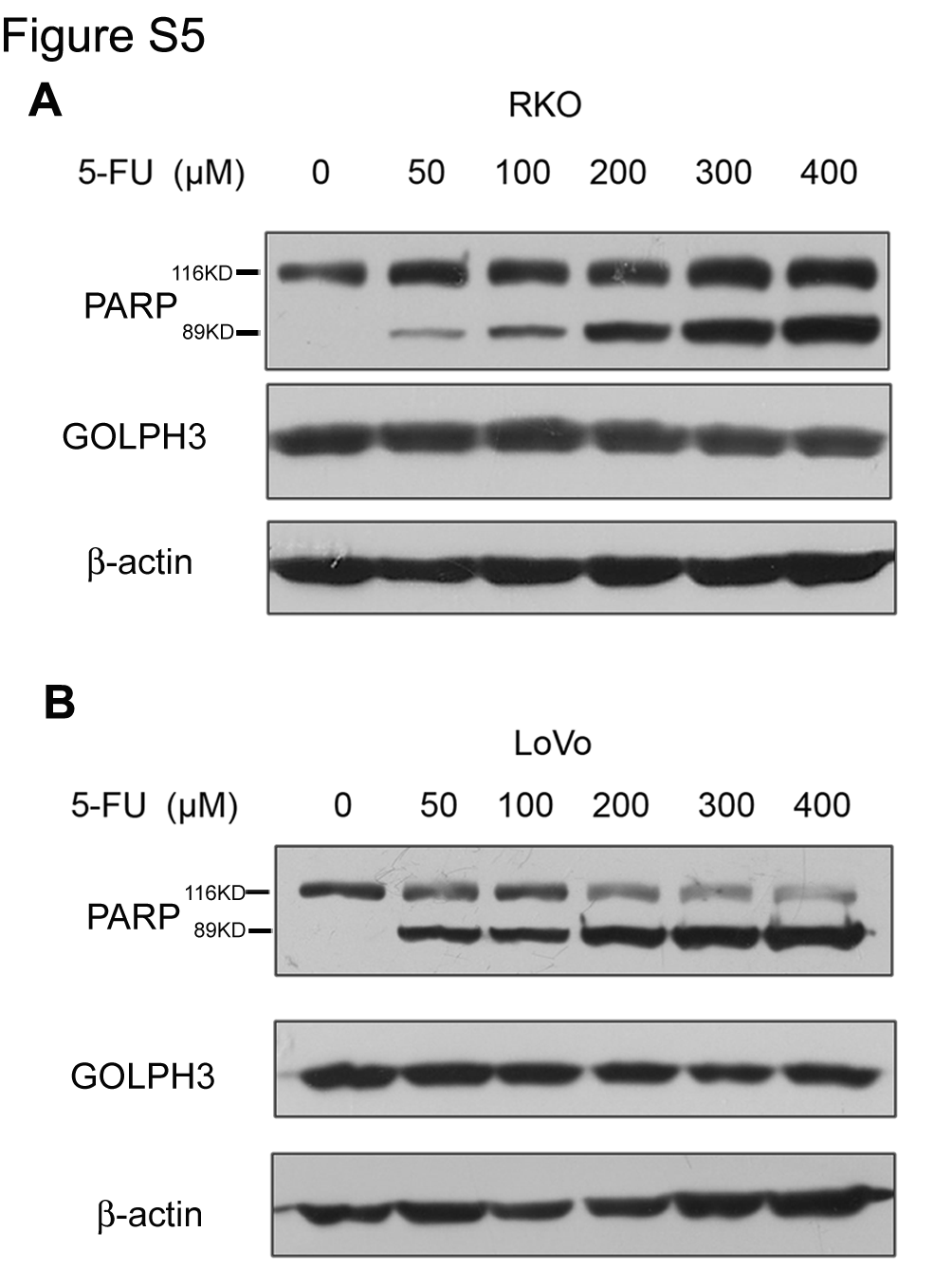

Supplement: Additional file 7: Figure S5 — 5-FU treatment does not affect the protein level of GOLPH3. GOLPH3 expression was determined by Western blot in RKO (A) and LoVo cells (B) treated with different concentrations of 5-FU. [file 1479-5876-12-15-S7.tiff]

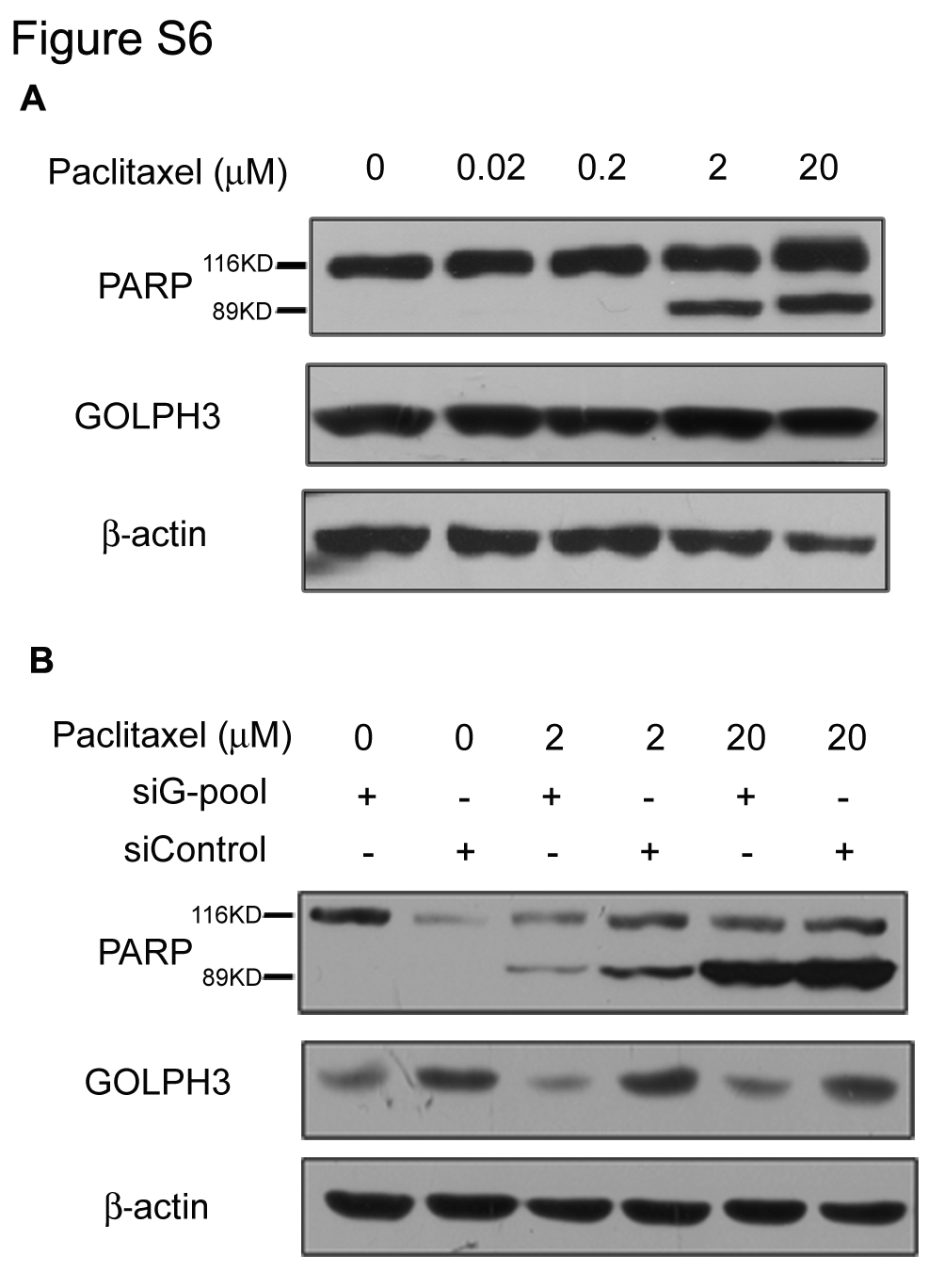

Supplement: Additional file 8: Figure S6 — The expression of GOLPH3 and cleaved PARP in RKO cells treated with paclitaxel. (A) Expression of GOLPH3 was determined by Western blot in RKO cells treated with various concentrations of paclitaxel for 48 h. Paclitaxel treatment does not affect GOLPH3 expression. (B) Knockdown of GOLPH3 reduced paclitaxel-induced apoptosis. The cleavage of PARP in paclitaxel treated GOLPH3-silencing and control cells was examined by Western blot. The results are representative of three independent experiments. [file 1479-5876-12-15-S8.tiff]
